# STING is a prognostic factor related to tumor necrosis, sarcomatoid dedifferentiation, and distant metastasis in clear cell renal cell carcinoma

**DOI:** 10.1007/s00428-023-03549-y

**Published:** 2023-04-29

**Authors:** Stefano Marletta, Anna Caliò, Giuseppe Bogina, Mimma Rizzo, Matteo Brunelli, Serena Pedron, Lisa Marcolini, Lavinia Stefanizzi, Stefano Gobbo, Alessandro Princiotta, Camillo Porta, Angela Pecoraro, Alessandro Antonelli, Guido Martignoni

**Affiliations:** 1 Department of Diagnostic and Public Health, Section of Pathology, University of Verona, Largo L. Scuro 10, 37134 Verona, Italy; 2 Department of Pathology, Pederzoli Hospital, Peschiera del Garda, Italy; 3 Department of Pathology, IRCCS Sacro Cuore Don Calabria Hospital, Negrar, Italy; 4 Division of Medical Oncology, A.O.U. Consorziale Policlinico di Bari, Bari, Italy; 5 Department of Translational Medicine, University of Ferrara, Ferrara, Italy; 6 Department of Urology, University of Verona, Verona, Italy; 7 Interdisciplinary Department of Medicine, University of Bari “A. Moro,”, Bari, Italy; 8 Department of Urology, San Luigi Gonzaga Hospital, University of Turin, Orbassano, Turin, Italy; 9 Department of Urology, Pederzoli Hospital, Peschiera del Garda, Italy

**Keywords:** Clear cell renal cell carcinomas, STING, Immunohistochemistry, Cancer immune infiltrate, Tumor-infiltrating lymphocytes, Prognosis

## Abstract

STING is a molecule involved in immune reactions against double-stranded DNA fragments, released in infective and neoplastic diseases, whose role in the interactions between immune and neoplastic cells in clear cell renal cell carcinoma has not been studied yet. We investigated the immunohistochemical expression of STING in a series of 146 clear-cell renal cell carcinomas and correlated it with the main pathological prognostic factors. Furthermore, tumoral inflammatory infiltrate was evaluated and studied for the subpopulations of lymphocytes. Expression of STING was observed in 36% (53/146) of the samples, more frequently in high-grade (G3–G4) tumors (48%,43/90) and recurrent/metastatic ones (75%, 24/32) than in low grade (G1–G2) and indolent neoplasms (16%, 9/55). STING staining correlated with parameters of aggressive behavior, including coagulative granular necrosis (*p* = 0.001), stage (*p* < 0.001), and development of metastases (*p* < 0.001). Among prognostic parameters, STING immune expression reached an independent statistical significance (*p* = 0.029) in multivariable analysis, along with the stage and the presence of coagulative granular necrosis. About tumor immune-environment, no significant statistical association has been demonstrated between tumor-infiltrating lymphocytes and STING. Our results provide novel insights regarding the role of STING in aggressive clear cell renal cell carcinomas, suggesting its adoption as a prognostic marker and a potentially targetable molecule for specific immunotherapies.

**Supplementary Information:**

The online version contains supplementary material available at 10.1007/s00428-023-03549-y.

## Introduction

Stimulator of interferon genes (STING), also known as transmembrane protein 173 (TMEM173), is a molecule physiologically placed on the endoplasmic reticulum which regulates several aspects of the immune response to intracellular double-stranded DNA fragments [1]. Namely, these latter ones prompt the enzyme cyclic GMP-AMP synthase (cGAS) to produce cyclic GMP-AMP which then binds two molecules of STING [2]. This causes STING to translocate to the Golgi network and, through interactions with transcriptional factors such as interferon regulatory factor 3 (IRF3) and nuclear factor kappa B (NFκb), to activate the transcription of key inflammatory genes, including IL-6, tumor necrosis factor (TNF), and interferon (IFN) α and β [3]. In humans, STING is mainly expressed by different types of immune cells, like T lymphocytes, macrophages, dendritic cells, and plasmacytoid dendritic cells, which activate its signaling pathway in response to double-stranded DNA molecules deriving from either exogenous or endogenous sources [4]. Thus, this not only explains why is STING involved in physiological inflammatory reactions against bacterial [5] and viral agents [6, 7] but also why it plays a significant role in regulating the complex immune response occurring in both autoimmune diseases, such as systemic lupus erythematosus [8] and rheumatoid arthritis [9], and, ultimately, cancer. Specifically, several studies have suggested that *STING* may act as either an oncogene or a tumor suppressor gene in some of the most common human cancers, such as colon [10], lung [11], and breast [12] carcinomas. As far as renal tumors are concerned, strong immunohistochemical expression of STING has been recently described in perivascular epithelioid cell (PEC) lesions of the kidney, while MiT family translocation renal cell carcinomas have been shown to stain negative for this marker [13]. Regarding other renal cell carcinomas, high STING mRNA levels and high immunohistochemical cytoplasmic expression of STING have been reported in renal medullary renal cell carcinoma [14], supporting further studies assessing the role of the cGAS-STING pathway in the immunotherapy of this tumor. Although in the last years, the role of the immune system in the biology of clear cell renal cell carcinoma has been extensively studied, no data regarding the cGAS-STING pathway are available. Therefore, in this study, we investigated STING immunohistochemical expression and its potential diagnostic and biological implications in a series of clear cell renal cell carcinomas.

## Methods

### Pathological features

One hundred forty-six clear cell renal cell carcinomas were retrieved from the files of participating institutions, including samples from both primary neoplasms and distant metastasis when available. Data were recorded from electronic health databases. All procedures performed in our study involving human participants received institutional review board approval and were in accordance with the ethical standards of the institutional and/or national research committee and with the declaration of Helsinki. All patients gave their written informed consent to diagnostic procedures and treatment according to institutional rules for everyday clinical practice and experimental evaluations on archival tissue. All slides were reviewed by three authors (S.M., A.C., G.M.). Regarding morphological features, each tumor was evaluated for the nucleolar grade according to the International Society of Urological Pathology (ISUP) and WHO 2022 classification, the state of surgical margins, the pTNM stage according to the 8th edition of the AJCC Cancer Staging Manual, the presence/absence of a sarcomatoid component, the percentage of coagulative-granular necrosis, and the presence/absence of tumor-infiltrating lymphocytes. As for the stage, tumors were gathered into three categories: stages I–II (low stage), stage III (intermediate stage), and stage IV (high stage). According to the distribution of tumor-infiltrating lymphocytes in or around the cancer cells, the inflammatory infiltrate was graded in three categories as suggested by previous studies [15, 16]: “desert” (tumor-infiltrating lymphocytes absent either within the tumor or its periphery), “excluded” (tumor-infiltrating lymphocytes accumulate around the tumor but without relevant infiltration within the lesion), and “inflamed” (tumor-infiltrating lymphocytes are intermixed with the neoplastic cells in the entire tumor area). Follow-up data on the development of distant metastases and/or local recurrence, defined as further tumor detection at the site of previously performed surgical procedures, were recorded from electronic health databases relying on the available radiological and pathological records.

### Immunohistochemistry

Sections from tissue blocks of all cases were immunohistochemically stained with STING (anti-TMEM173; clone SP338, dilution 1:150; Abcam, UK). Heat-induced antigen retrieval for STING was performed using a microwave oven and 0.01mol/L of citrate buffer, pH 8.0, for 30 min. Moreover, tumors defined as “excluded” and “inflamed” by hematoxylin and eosin were investigated for the specific composition of different lymphocytes subpopulations, according to the different percentages of expression of the following antibodies: CD3 (clone PS1, dilution 1:200, LEICA), CD20 (clone L26, prediluted, NOVOCASTRA), CD4 (clone 4B12, dilution 1.150, LEICA), CD8 (clone 29S, dilution 1:20, LEICA), and FOXP3 (clone 221D/D3, dilution 1:200, SEROTEC). All samples were processed using a sensitive “Bond Polymer Refine” detection system in an automated bond immunohistochemistry instrument (Leica Biosystems, Germany). Sections incubated without the primary antibody served as a negative control. Cytoplasmic and membranous labeling for the STING was recorded by combining the percentage of positive cells (0–100%) multiplied by staining intensity (0, 1+, 2+, and 3+) to obtain an overall H-score (0-300).

### Statistical analysis

For statistical analysis, data were imported and performed using STATA/IC for Windows version 14.0. Differences in distribution and frequency between clinicopathologic characteristics, distribution of tumor-infiltrating lymphocytes, and STING expression were analyzed using the *χ*^2^ test, distinguishing between low (H-score ≤ 5) and high (H-score > 5) levels of STING. As for the ISUP/WHO nucleolar grade, while G1 and G2 were collected together, G3 and G4 were considered separately. In detail, as suggested by previous works [17], the statistical analysis was conducted both by gathering G3 neoplasms displaying foci of necrosis with G4 ones as well as distinguishing G3 and G4 tumors individually regardless of the presence of necrosis.

Time to recurrence was calculated from the date of surgery to the date of recurrence or metastasis. Patients alive and not relapsing were censored at the date of their last follow-up visit. The cumulative incidence of time to recurrence in the groups was described by the Kaplan–Meier method and compared with the use of the log-rank test. The Cox proportional hazard regression model was used to evaluate the associations between clinicopathological factors and clinical outcomes. A two-sided *p*-value <0.05 was considered statistically significant.

## Results

### Clinical features

Forty-one patients were females, and one hundred-three were males (F:M ratio 1:2,5). Age at diagnosis ranged from 32 to 91 years (mean 64, median 65). At the time of the diagnosis, according to the pTNM classification, 66 patients were considered as low stage (I-II), 65 as intermediate stage (III), and 12 as high stage (IV); no data was available for the remaining three of them. Follow-up was available for 139 patients, ranging from 1 to 149 months (mean 41, median 9). Thirty-two patients displayed aggressive clinical behavior developing local recurrence (3 cases) and/or distant metastases (29 cases) after 34 months on average (range from 1 and 129 months, median 6) since the diagnosis of the primary renal tumor. The site of metastatic disease was variable involving abdominal lymph nodes (5 cases), the lungs (21 cases), the pancreas (8 cases), the adrenal glands (9 cases), the brain (4 cases), the liver (3 cases), the thyroid gland (1 case), the urinary bladder (1 case), the colon (1 case), the peritoneum (1 case), and the vagina (1 case). Microscopic involvement of surgical resection margins (R1) was only detected in 3 of the samples (cases 76, 78, and 84), none of them developing local recurrence nor distant metastases on follow-up. Regarding systemic therapies in metastatic patients, most of them were given tyrosine-kinase inhibitors (TKI), while in one case (patient 71), a combination between a TKI (axitinib) and an immune-checkpoint inhibitor (ICI) (pembrolizumab) was administered. One of the patients (case 31) simultaneously underwent pancreatic resection for a neuroendocrine tumor, while another one (case 15), during follow-up, was diagnosed with colon cancer and afterward treated for it. Moreover, following surgical treatment of renal cell carcinoma, one patient was diagnosed with both prostatic adenocarcinoma and diffuse large B cell lymphoma (case 119), whereas another suffered from infiltrating high-grade bladder urothelial carcinoma with skull metastases (case 89).

### Pathological and immunohistochemical findings

One hundred-one patients underwent radical nephrectomy, while in the other 43 cases, the neoplasms were removed by partial nephrectomy. In two specimens, two different clear cell renal carcinomas were found within the same kidney (cases 112 and 137). The tumors ranged in size from 1.2 to 17 cm (mean 6, median 5). As for the nucleolar grade, according to the ISUP/WHO 2022 system, 55 tumors were classified as low-grade (G1–G2) while 90 of them as high-grade neoplasms, namely, 56 G3 and 34 G4. In twelve samples, all belonging to the high-grade (G4) group, a variable amount of sarcomatoid dedifferentiation was observed, being of rhabdoid type in 4 of them. In one case, information regarding the nucleolar grade of the primary renal tumor was not available, as only slides from a pancreatic metastasis were retrieved (case 37). All but three patients who developed metastatic diseases were affected by high-grade primary tumors (G3–G4). Foci of coagulative granular necrosis were detected in 54 tumors, 98% of which (53/54) displayed high nucleolar grade (G3–G4). Tumor-associated inflammatory infiltrate was scored as desert in 72% (105/146) and as either “excluded” or “inflamed” in 28% (41/146) of the samples, the majority of these latter (80%, 33/41) belonging to the high grade (G3–G4) group: namely, all but three of the 25 “inflamed” cases (88%) were represented by morphologically aggressive tumors (G3–G4), while the 16 “excluded” samples with peripheral limited tumor-infiltrating lymphocytes’ accumulation were slightly more evenly distributed between low-grade (31%) and high-grade (69%) neoplasms.

**Fig. 1:**
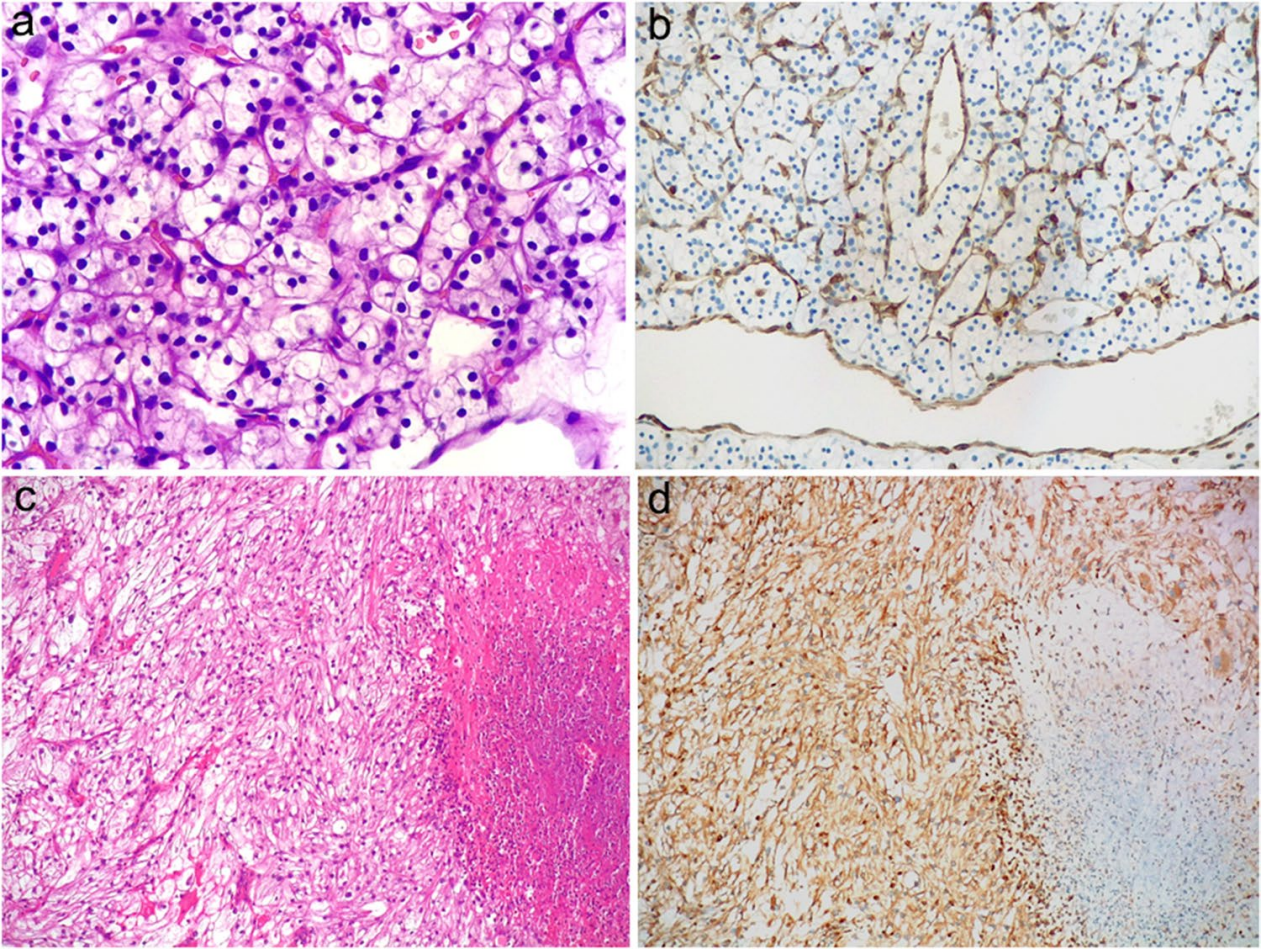
Low-grade clear cell renal cell carcinoma (**a**) staining negative for STING in neoplastic cells; conversely, endothelial cells of intratumoral capillaries labeled positive for STING (**b**). High-grade clear cell renal cell carcinoma (**c**) showing strong and diffuse immunohistochemical expression of STING, apart from a necrotic area on the left (**d**) (original magnification 100× (**c** and **d**), 200× (**b**) and 400× (a))

**Fig. 2:**
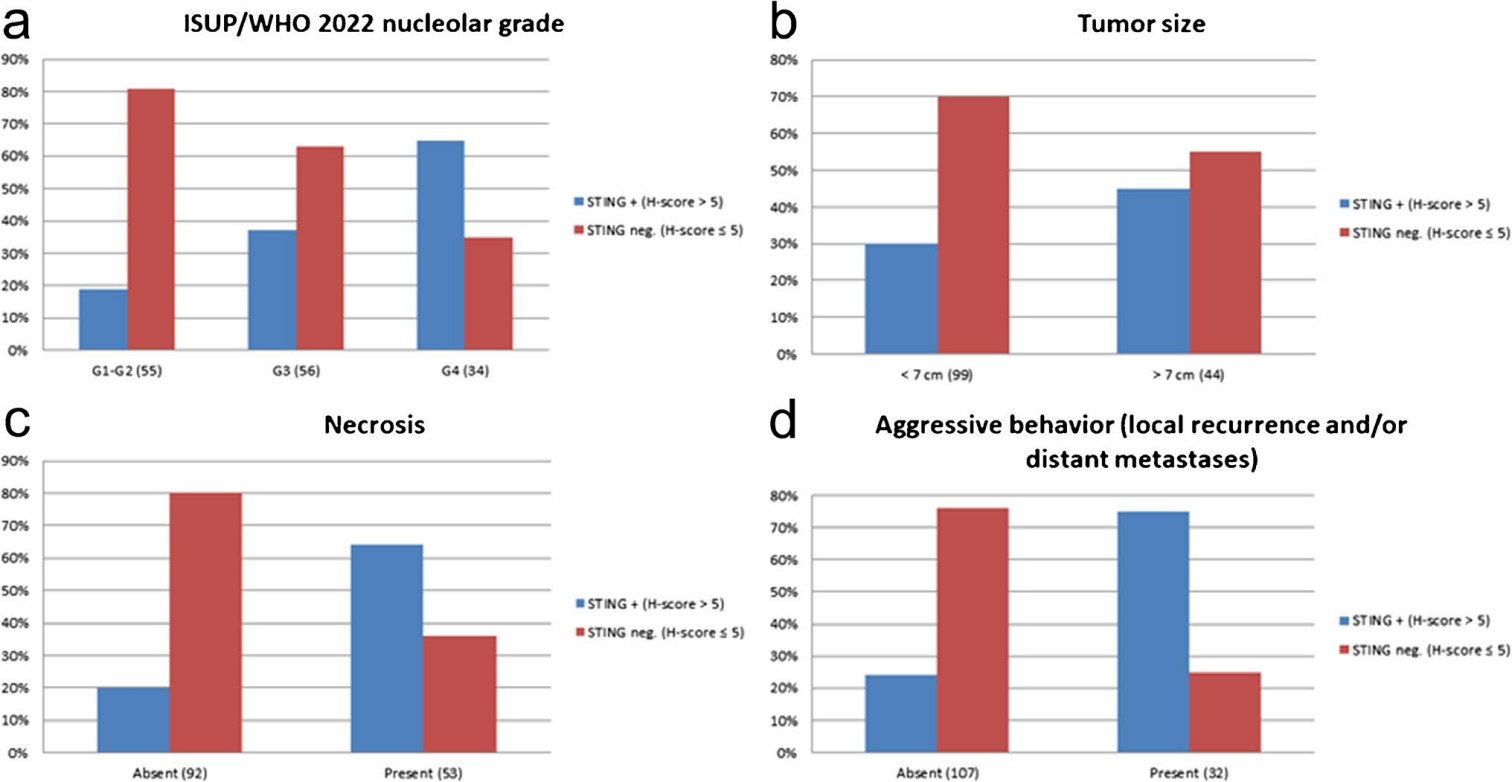
Charts showing the correlation between STING immunohistochemical expression and ISUP/WHO 2022 grading, tumor size, presence of coagulative granular necrosis, and development of an aggressive clinical behavior

Membranous/cytoplasmic immunohistochemical expression of STING, considered as an H-score > 5 (spanning from 5 to 290), was found in 53 of 146 samples (36%), including 9 of the 55 low-grade (G1–G2) tumors (16%), and 43 of the 90 high grade (G3–G4) carcinomas (48%) (Fig. 1), and one pancreatic metastasis, which represented the only available specimen from the corresponding case (patient 37). As for high-grade (G3–G4) tumors, positive immunolabelling for STING (H-score > 5) was noticed in 21 of the 56 G3 neoplasms (37%) and 22 of the 34 G4 ones (65%). In addition, 58% (7/12) of the tumors showing a sarcomatoid component stained positive for STING, with an overall H-score ranging from 10 to 290. Furthermore, 64% (34/53) of the tumors displaying variable foci of necrosis were labeled positive for STING (H-score spanning from 10 to 290). About clinically aggressive tumors, immunolabelling for STING was recorded in 24 of 32 neoplasms (75%), variably observed in primary tumors and metastatic lesions, with an H-score ranging from 10 to 250 (Fig. 2).

**Fig. 3:**
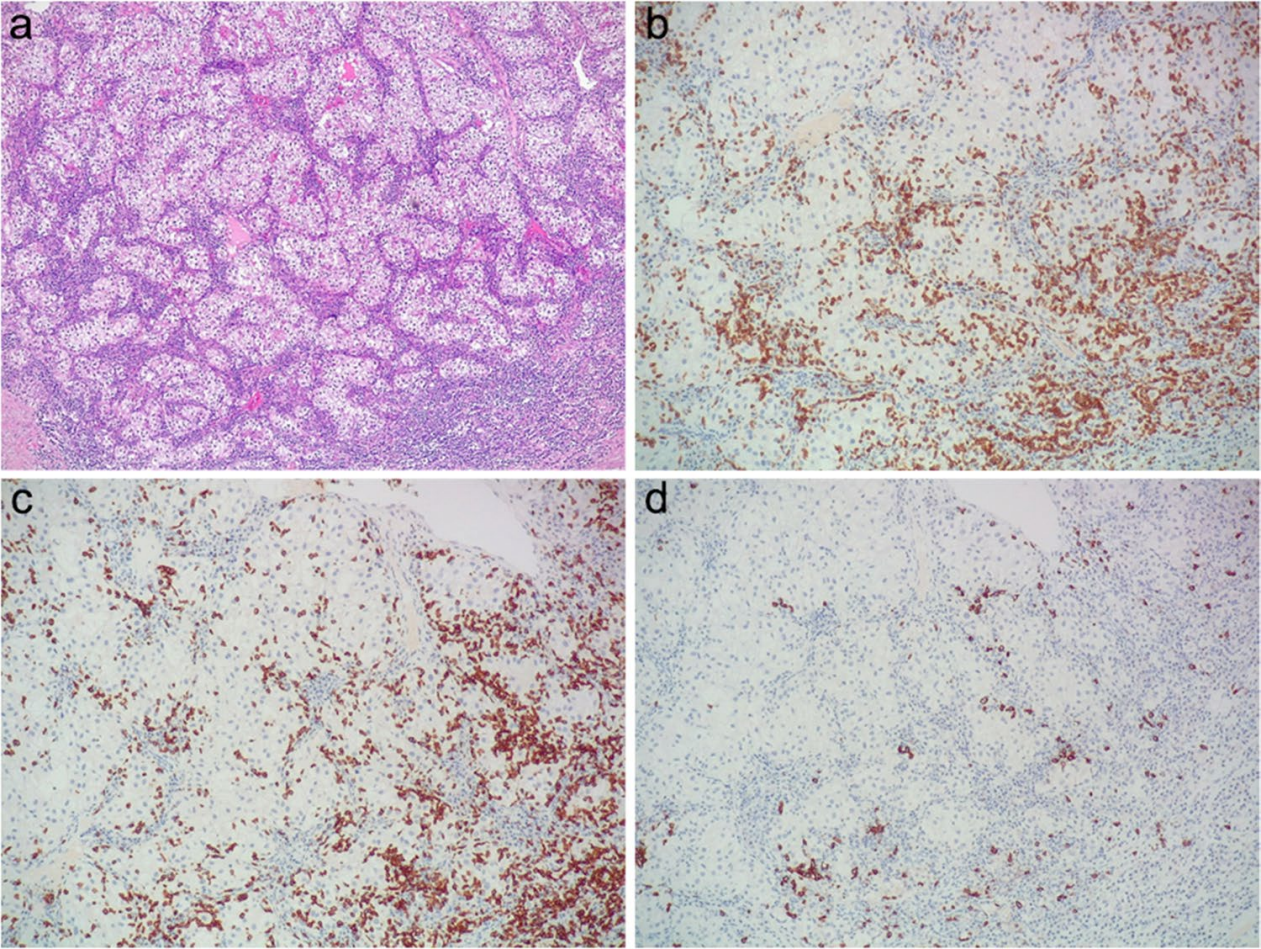
An example of “inflamed” clear cell renal cell carcinoma (**a**), showing a marked peritumoral and intratumoral infiltrate enriched with CD3^+^ T lymphocytes (**b**), especially of the CD8^+^ T-cytotoxic subset (**c**), and only a few CD20^+^ B lymphocytes (**d**) (original magnification 50× (**a**), 100× (**b**, **c**, **d**))

As for the specific composition of tumor-infiltrating lymphocytes, generally, both “excluded” and “inflamed” samples showed a prevalent CD3^+^ T-lymphocytes related response, variably making up from 50 to 95% of overall immune cells, while CD20^+^ B-lymphocytes accounted for a restricted proportion of all the inflammatory cells. Just a few scattered FOXP3^+^ T-regulatory lymphocytes were detected in each of the samples. In detail, a CD8^+^ T-cytotoxic response was significantly prevalent in those tumors effaced by a remarkable inflammatory infiltrate (Fig. 3). Conversely, “excluded” cases not only revealed a less prominent T-lymphocytes response than “inflamed” ones, with a mean higher amount of CD20^+^ B-lymphocytes, but they also showed a slightly more homogenous distribution of CD8^+^ T-cytotoxic lymphocytes and CD4^+^ T-helper lymphocytes.

**Fig. 4:**
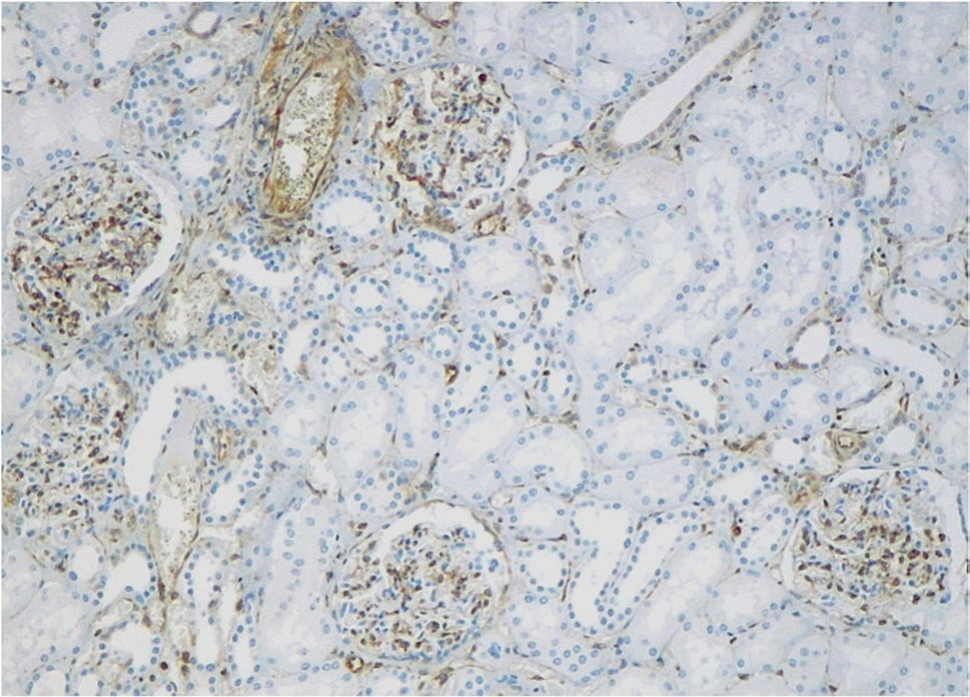
Normal renal parenchyma displaying variable staining for STING in the endothelial cells of the vascular pole of glomeruli and blood vessels, as well as in these latter ones’ smooth muscle layer and in interstitial stromal cells (original magnification 100×)

In normal renal parenchyma, cytoplasmic STING expression was found in the cells of the vascular pole of glomeruli, likely the juxtaglomerular apparatus, in the endothelial cells of glomerular capillaries, and the interstitial stromal cells (Fig. 4). As for renal tubules, variable staining of STING was seen in the collecting ducts and the distal tubules, whereas the proximal tubules were constantly negative. Positive labeling for STING was found in endothelial cells and the smooth muscle cells of the vessels’ wall within both normal parenchyma of the kidney and tumor lesions as well. Finally, a variable proportion of peritumoral and intratumoral lymphocytes in “excluded” and “inflamed” neoplasms, different from case to case, showed immune expression of STING.

Clinical, pathological, and immunohistochemical features of the present series are summarized in Table 1 and further specifically detailed in supplementary Table S1.

**Table 1:** Summary of clinical-pathological features of renal cell carcinomas of the present series

Parameter	No. of cases (%)	Parameter	No. of cases (%)
Sex		Margin status	
F	41 (28)	R0	142 (98)
M	103 (72)	R1	3 (2)
Age	32–91 y.o., median 65	Necrosis	
Size		Absent	92 (63)
≤ 7 cm	99 (69)	Present ≤ 10%	33 (23)
> 7 cm	44 (31)	Present > 10%	20 (14)
Laterality		STING IHC	
Right	70 (48)	Negative	90 (62)
Left	75 (52)	Low (≤ 10%)	18 (12)
ISUP/WHO Grading		High (> 10%)	38 (26)
1	9 (6)	H-score	
2	46 (32)	≤ 5	93 (64)
3	56 (39)	> 5	53 (36)
4	34 (23)	Inflammatory infiltrate	
pTNM stage		Desert	105 (72)
I	59 (40)	Excluded	16 (11)
II	4 (3)	Inflamed	25 (17)
III	55 (38)	Follow up, months	1–149 (mean 41)
IV	28 (19)	Clinical behavior	
Surgery		NED	107 (77)
Radical nephrectomy	101 (70)	Local recurrence	3 (2)
Partial nephrectomy	43 (30)	Metastases	29 (21)

*NED* no evidence of disease

### Statistical correlation between STING immunohistochemical expression, clinicopathological variables, and distribution of tumor-infiltrating lymphocytes

Using the chi-square test, a statistically relevant association was observed between STING positive immunohistochemical staining stratified according to the H-score and male sex (*p* = 0.024), high pTNM stage (*p* < 0.001), presence of necrosis (*p* = 0.001), and high ISUP/WHO 2022 nucleolar grade, this latter both considering G3 tumors whether (*p* < 0.001) or not (*p* < 0.001) they showed foci of coagulative necrosis. As for inflammation, no association was found with STING expression, neither when considering “desert” plus “excluded” versus “inflamed” tumors (*p* = 0.073) nor when matching “desert” versus “excluded” plus “inflamed” neoplasms (*p* = 0.669).

**Fig. 5:**
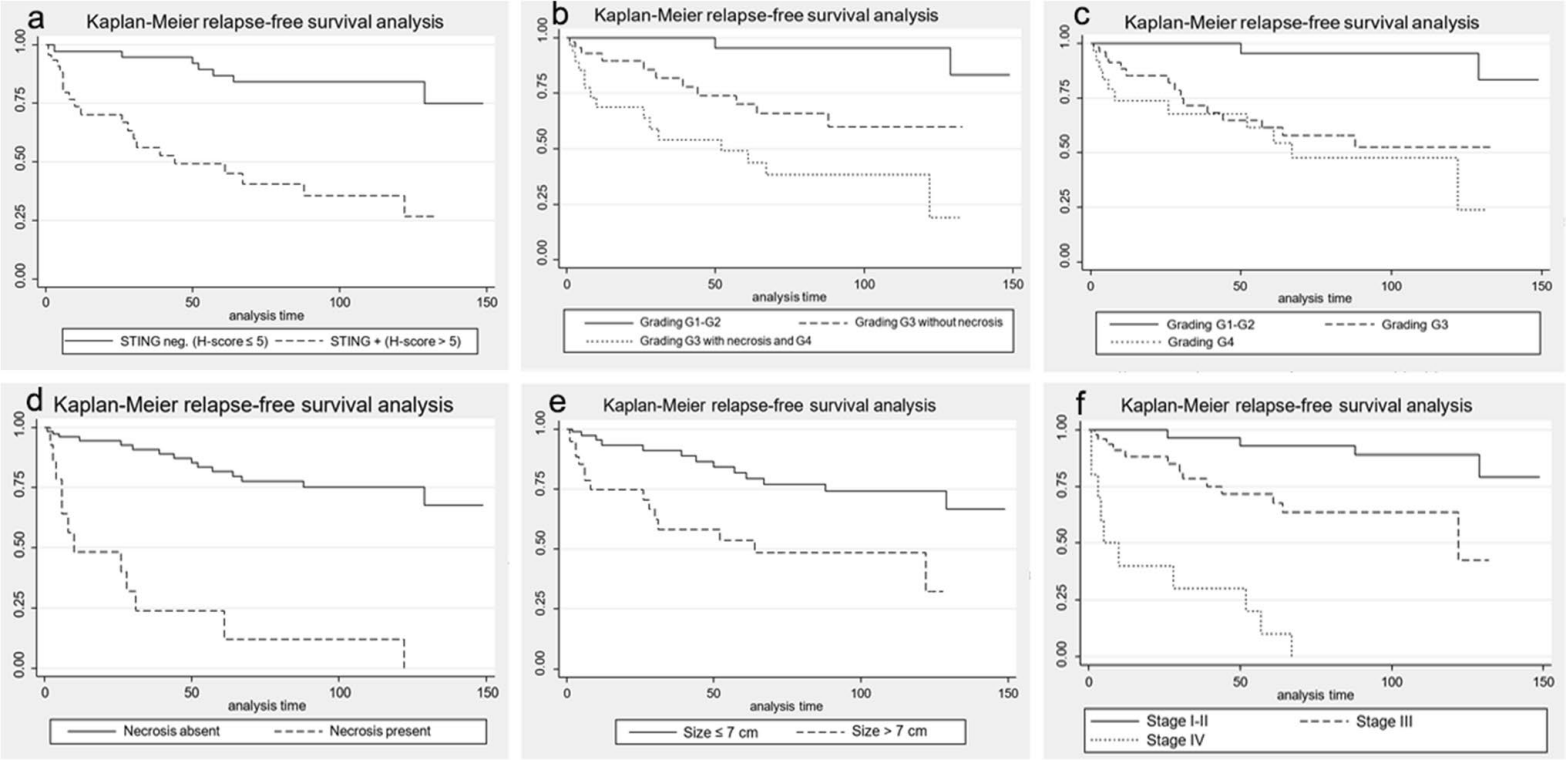
Kaplan-Meier curves analyzing the association between the development of recurrence or distant metastases and STING immunohistochemical expression (**a**), ISUP/WHO 2022 nucleolar grading (**b**, **c**), presence/absence of necrosis (**d**), tumor size (**e**), and pTNM tumor stage (**f**)

Kaplan-Meier statistics demonstrated that STING expression was associated with a higher probability of disease recurrence/metastases (*p* < 0.001), as well as high nucleolar grade (*p* = 0.0004 and *p* < 0.001), presence of coagulative granular necrosis (*p* < 0.0001), high pTNM stage (*p* < 0.001), and large tumor size (*p* = 0.0007) (Fig. 5). When a Cox proportional hazard regression model was employed for carrying a multivariable analysis among STING immunohistochemical expression, tumor size, nucleolar grade, coagulative granular necrosis, and clinical outcome, immunolabeling for such marker (*p* = 0.029), presence of necrosis (*p* = 0.026), and pTNM stage (*p* = 0.002) reached a statistical independent significance (Table 2).

**Table 2:** Multivariable analysis concerning the main clinicopathological parameters and STING expression

Variable	HR (95% CI)	*p* value
Surgery	3.18 (0.38–26.45)	0.283
ISUP/WHO Grading	0.58 (0.24–1.37)	0.219
Necrosis	3.24 (1.14–9.15)	**0.026**
pTNM stage	5.50 (2.40–12.57)	**< 0.001**
STING	3.37 (1.13–10.04)	**0.029**

*HR* hazard ratio, *CI* confidence interval

Statistically relevant results (*p* < 0.05) are highlighted in bold

## Discussion

Despite the advances obtained regarding tumor biology and the development of more efficient therapeutic agents, clear cell renal carcinoma and, generally, kidney cancer still constitute a health problem of major concern in Western countries. In fact, during their lifetime, more than half of clear cell renal cell carcinoma patients develop synchronous or metachronous metastases and are then currently treated with combinations of ICIs and TKIs [18].

STING is a molecule involved in the inflammatory reaction to microbes, like viruses and bacteria, whose function has been recently shown to potentially modulate cancer immune response in several kinds of human malignancies. On these bases, in the present study, we have evaluated the immunohistochemical expression of STING in a broad series of clear cell renal cell carcinomas with different clinical behavior. We have found an increased immunohistochemical expression of STING in 75% (24/32) of metastatic or locally aggressive clear cell renal cell carcinomas in contrast to clinically indolent ones (23%, 26 of 114 tumors). Not only STING immunohistochemical expression in our series was significantly correlated with the development of recurrence or distant metastasis (*p* < 0.001), but it also reached a statistically independent meaning (*p* = 0.029) in multivariable analysis, along with pTNM stage and the presence of coagulative granular necrosis. This finding suggests its employment as a further useful parameter of adverse clinical behavior, along with other ones already known for harboring prognostic value, including tumor size, ISUP/WHO nucleolar grading, and coagulative granular necrosis [19–22]. Interestingly, a wide amount (18 of 26 cases, 69%) of not-metastatic neoplasms positive for STING variably showed either high nucleolar grade (G3–G4), coagulative granular necrosis, or both. It is possible to hypothesize that these tumors are likely to harbor metastatic potential and then spread subsequently to other organs with a longer follow-up.

A possible explanation of STING expression in aggressive clear cell renal cell carcinomas may be its relationship with abnormal oxidative stress. It has been stated that a genomic signature characterized by overexpression of oxidative stress-related genes is significantly related to the probability of developing lymph nodes metastasis in clear cell renal cell carcinomas [23, 24]. Similarly, abnormal oxidative stress induces persistent DNA damage, alteration of function of proteins of the nuclear membrane and thus of its integrity [25], the release of blebs containing genomic damaged double-stranded DNA from the cytoplasm [26], and, ultimately, triggering of the cGAS-STING machine [2].

Since the cGAS-STING pathway has a role in immune-regulatory signaling, we have also evaluated the inflammatory infiltrate in our series and its correlation with STING immunohistochemical expression. Tumor-associated inflammatory infiltrate was observed in 28% of the tumors, most of them with high nucleolar grade (G3–G4). Interestingly, all but three of the 25 “inflamed” cases were represented by morphologically aggressive tumors and the tumor-associated inflammatory infiltrate was characterized by a CD8^+^ T-cytotoxic response. Conversely, “excluded” tumors not only revealed a less prominent T-lymphocytes response than “inflamed” ones, with a mean higher amount of CD20^+^ B-lymphocytes, but they also showed a slightly more homogenous distribution of CD8^+^ T-cytotoxic lymphocytes and CD4^+^ T-helper lymphocytes.

When analyzing the correlation between STING expression and tumor-associated infiltrate, no significant statistical association emerged from our study. In detail, immunohistochemical expression of STING did not correlate with tumor-infiltrating lymphocytes neither when matching “desert” plus “excluded” versus “inflamed” tumors (*p* = 0.073) nor when performing statistical analysis of “desert” neoplasms versus “excluded” plus “inflamed” ones (*p* = 0.669). A possible explanation of this finding may be linked to a different result of the activation of the cGAS-STING pathway in biologically aggressive neoplasms [1]. While low activation levels of this pathway seem to sustain a T-cytotoxic antitumoral effect via a type I interferons-mediated response [27, 28], chronic persistence of DNA damage and release of double-stranded DNA particles with subsequent c-GAS-STING activation may be responsible for a down-regulation of a cancer-related immune response. This likely occurs through the production of immunosuppressive cytokines and the induction of a senescence-associated secretory phenotype [29, 30], which ultimately leads to the proliferation of tumor cells and the development of metastasis [12, 31]. Namely, recent elegant studies on triple-negative breast cancer lines and animal models have shown that chromosomal instability in neoplastic cells may trigger the cGAS-STING pathway via the formation of micronuclear vesicles [32]. Through a non-canonical NF-κB activation, this could cause IL6 expression to increase and then to up-regulate the STAT3-dependent signaling, ultimately leading to cancer cell survival and silencing of tumor-related inflammatory response. Such findings contribute to explaining why the loss of function of genes of the *cGAS-STING* pathway is only rarely encountered in chromosomally unstable cancers. Similarly, it has been recently reported that high IL6 levels are associated with poorer outcomes and lower treatment response in renal cell carcinoma patients [33]. Thus, the absence of a correlation between STING immunohistochemical expression and the increasing amount of CD8^+^ T-cytotoxic lymphocytes intratumoral inflammatory infiltrate in our series may further strengthen the hypothesis of an immune-suppressive role for this pathway in morphologically aggressive renal cell carcinomas. The reduced anti-tumoral immune response in these settings of tumors, related to the activation of the STING pathway, may help these neoplasms to fully gain a metastatic potential.

From a therapeutic point of view, it has been demonstrated that patients with higher levels of tumor-infiltrating lymphocytes are more likely to respond to immune checkpoint modulators [34], which are nowadays a milestone for advanced renal cancer treatment [35–37]. Blockage of the STING pathway in morphologically aggressive renal cell carcinomas prevents local progression and metastasis development, eliciting a stronger antitumoral response [38]. Accordingly, in the aforementioned study on triple-negative breast cancer lines, silencing of the cGAS-STING-IL6-STAT3 axis by tocilizumab, a monoclonal antibody inhibiting the IL6-receptor, selectively reduced proliferation and viability of cells carrying high chromosomal instability. No significant effects were instead noted in those lines without in vitro induced chromosomal instability nor in normal epithelial breast cells [39]. Considering that increased IL6 rates have been associated with a severe prognosis in renal cell carcinoma patients [33], these results open new insights about the employment of tocilizumab or other related molecules for improving the outcome of such patients, potentially enhancing the effect of the other currently available immunotherapies. In this scenario, immunohistochemical expression of STING could be adopted as a predictive efficacy biomarker for identifying which patients affected by metastatic or morphologically aggressive tumors may benefit from immunotherapy.

In conclusion, in our study, we have demonstrated a significant association between immunohistochemical expression of STING and high-grade (G3–G4) metastatic clear cell renal cell carcinomas, in contrast to organ-confined low-grade (G1–G2) lesions which have less frequently expressed this marker. Along with pTNM stage and the presence of coagulative granular necrosis, STING expression reached a statistically independent significance (*p* = 0.036) in multivariable analysis among prognostic factors. Despite further studies being warranted for investigating its specific function in clear cell renal cell carcinomas, our data suggest a prognostic role for this marker. Moreover, experimental observations of an immunoregulative effect of the activation of the STING by aggressive tumors advocate for the potential therapeutic use of modulators of the pathway of this molecule as well, especially along with immune-checkpoints modulators.

## Supplementary information

ESM 1: **Table S1.** Clinical-pathological features and STING expression of renal cell carcinomas of the present series. (DOCX 61 kb)

## Data Availability

All data generated or analyzed during this study are included in this published article (and its supplementary information files).
