# Supplementary material for: STING is a prognostic factor related to tumor necrosis, sarcomatoid dedifferentiation, and distant metastasis in clear cell renal cell carcinoma
Source: Virchows Arch. 2023 Apr 29;483(1):87–96. doi: 10.1007/s00428-023-03549-y (PMC10326155; doi:10.1007/s00428-023-03549-y)
Supplement: Supplementary file 1 — Table S1. Clinical-pathological features and STING expression of renal cell carcinomas of the present series. (DOCX 61 kb) [file 428_2023_3549_MOESM1_ESM.docx]

**Table S1.** Clinical-pathological features and STING expression of renal cell carcinomas of the present series.

| **Case N.** | **Sex** | **Age** | **Size/Laterality** | **pTNM** | **Grade** | **Surgery** | **Margins** | **Necrosis** | **STING** | **H-Score** | **TILs** | **Follow up (months)** |
| --- | --- | --- | --- | --- | --- | --- | --- | --- | --- | --- | --- | --- |
| 1 | M | 65 | 5 cm/L | pT1bNxMx | 2 | R.N. | R0 | Absent | Neg. | 0 | Desert | 147 NED |
| 2 | F | 42 | 5 cm/R | pT1bNxMx | 1 | R.N. | R0 | Absent | Neg. | 0 | Desert | 149 NED |
| 3 | M | 45 | 9 cm/L | pT2aNxMx | 2 | R.N. | R0 | Absent | 30% + | 55 | Desert | 128 NED |
| 4 | F | 75 | 6 cm/R | pT3aNxMx | 4 | R.N. | R0 | 5% | Neg. | 0 | Desert | 132 NED |
| 5 | F | 75 | 1,8 cm/L | pT1aNxMx | 3 | P.N. | R0 | Absent | Neg. | 0 | Inflamed | 118 NED |
| 6 | F | 44 | 4 cm/L | pT1aNxMx | 2 | P.N. | R0 | Absent | Neg. | 0 | Desert | 146 NED |
| 7 | M | 69 | 3 cm/L | pT1aNxMx | 2 | P.N. | R0 | Absent | Neg. | 0 | Excluded | 89 NED |
| 8 | M | 75 | 6 cm/L | pT1bNxMx | 2 | P.N. | R0 | Absent | Neg. | 0 | Desert | 140 NED |
| 9 | M | 46 | 2,8 cm/R | pT1aNxMx | 2 | P.N. | R0 | 1% | 30% + | 45 | Desert | 127 NED |
| 10 | M | 68 | 3,8 cm/L | pT1aNxMx | 3 | P.N. | R0 | Absent | 20% + | 25 | Desert | 133 NED |
| 11 | M | 65 | 3 cm/R | pT1aNxMx | 2 | R.N. | R0 | Absent | Neg. | 0 | Desert | 130 NED |
| 12 | M | 66 | 1,8 cm/R | pT1aNxMx | 2 | P.N. | R0 | Absent | Neg. | 0 | Desert | 131 NED |
| 13 | M | 67 | 5 cm/R | pT3aNxMx | 3 | P.N. | R0 | Absent | Neg. | 0 | Desert | 112 NED |
| 14 | M | 58 | 6 cm/R | pT3aNxMx | 3 | R.N. | R0 | 1% | Neg. | 0 | Desert | 87 NED |
| 15 | F | 68 | 2,7 cm/R | pT1aNxMx | 2 | R.N. | R0 | Absent | Neg. | 0 | Excluded | 115 Colon cancer on F.U. |
| 16 | M | 56 | 3,5 cm/R | pT1aNxMx | 2 | P.N. | R0 | Absent | 50% + | 100 | Desert | 62 NED |
| 17 | M | 70 | 3,5 cm/R | pT3aNxMx | 3 | R.N. | R0 | Absent | Neg. | 0 | Desert | 92 NED |
| 18 | F | 71 | 3 cm/R | pT1aNxMx | 2 | R.N. | R0 | Absent | Neg. | 0 | Desert | 116 NED |
| 19 | M | 78 | 2 cm/L | pT1aNxMx | 1 | P.N. | R0 | Absent | Neg. | 0 | Desert | 109 NED |
| 20 | M | 80 | 7 cm/R | pT1bNxMx | 3 | R.N. | R0 | Absent | Neg. | 0 | Excluded | 98 NED |
| 21 | F | 58 | 3 cm/L | pT1aNxMx | 2 | P.N. | R0 | Absent | Neg. | 0 | Excluded | 108 NED |
| 22 | M | 69 | 3,8 cm/R | pT1aNxMx | 4* | R.N. | R0 | 5% | 20% + | 60 | Inflamed | 109 NED |
| 23 | M | 53 | 4,2 cm/R | pT3aNxMx | 4 | R.N. | R0 | 10% | 10% + | 10 | Desert | 101 NED |
| 24 | M | 77 | 10 cm/L | pT3aNxMx | 3 | R.N. | R0 | 5% | 70% + | 190 | Desert | 58 NED |
| 25 | M | 65 | 2,5 cm/L | pT1aNxMx | 2 | P.N. | R0 | Absent | Neg. | 0 | Excluded | 106 NED |
| 26 | M | 70 | 4 cm/L | pT1aNxMx | 3 | P.N. | R0 | Absent | Neg. | 0 | Desert | 106 NED |
| 27 | M | 63 | 5,5 cm/R | pT3aNxMx | 3 | R.N. | R0 | Absent | Neg. | 0 | Inflamed | 104 NED |
| 28 | M | 53 | 5 cm/R | pT1bNxMx | 2 | R.N. | R0 | Absent | Neg. | 0 | Desert | 129 NED |
| 29 | M | 59 | 1,8 cm/R | pT1aNxMx | 2 | P.N. | R0 | Absent | Neg. | 0 | Desert | N.A. |
| 30 | F | 48 | 10 cm/R | pT3aNxMx | 4 | R.N. | R0 | 30% | Neg. | 0 | Excluded | N.A. |
| 31 | M | 60 | 7,5 cm/L | pT3aNxMx | 4* | R.N. | R0 | 5% | Neg. | 0 | Desert | N.A. (synchronous pancreatic neuroendocrine tumor) |
| 32 | M | 77 | N.A. | pTxNxM1 | 4 | R.N. | R0 | 20% | 100% + | 290 | Desert | AWD (adrenal gland, pancreas, lung and thyroid) |
| 33 | M | 53 | N.A. | pTxNxM1 | 2 | R.N. | R0 | Absent | 30% + | 45 | Desert | AWD (adrenal gland and pancreas) |
| 34 | M | 69 | 4 cm/R | pT3aNxMx | 3 | R.N. | R0 | 5% | 10% + | 15 | Excluded | 44 AWD (lung, brain, lymph nodes and adrenal gland) |
| 35a | M | 54 | 7 cm/L | pT3aNxM1 | 3 | R.N. | R0 | 5% | 70% + | 150 | Desert | AWD (lung) |
| 35b | M | 54 | N.A. | pT3aNxM1 | N.A. | N.A. | N.A. | N.A. | 10% + | 10 | N.A. | AWD (lung) |
| 36 | M | 75 | 11 cm/L | pTxNxM1 | 4 | R.N./colic biopsy | R0 | 20% | 60% + | 180 | Desert | 6 AWD (colon, pancreas, lung and liver) |
| 37 | M | 71 | N.A./R | pTxNxM1 | N.A. | R.N./metastasectomy | R0 | N.A. | 30% + | 30 | Desert | 6 AWD (pancreas) |
| 38 | F | 49 | 5 cm/R | pT1bNxMx | 2 | R.N. | R0 | Absent | Neg. | 0 | Desert | 129 AWD (pancreas) |
| 39 | M | 74 | 8 cm/R | pT3aNxMx | 4* | R.N. | R0 | 40% | 10% + | 15 | Desert | 122 AWD (pancreas, lymph nodes and lung) |
| 40 | M | 66 | 6,5 cm/R | pT3aNxM1 | 4 | R.N. | R0 | 5% | 70% + | 200 | Inflamed | 67 AWD (lung) |
| 41 | M | 67 | 8 cm/R | pT3aNxM1 | 4* | R.N. | R0 | 5% | 5% + | 5 | Desert | 52 AWD (lung) |
| 42 | M | 66 | 5 cm/L | pT1bNxM1 | 3 | R.N. | R0 | 1% | 50% + | 120 | Inflamed | 88 AWD (lung, renal, liver, lymph nodes and adrenal gland) |
| 43 | M | 61 | 8,5 cm/L | pT3aNxMx | 3 | R.N. | R0 | 40% | 70% + | 180 | Excluded | 28 AWD (lung and brain) |
| 44 | M | 73 | 6,5 cm/R | pT3aNxM1 | 3 | R.N. | R0 | 1% | Neg. | 0 | Excluded | 57 AWD (pancreas and lung) |
| 45 | F | 52 | 3,2 cm/L | pT1aNxMx | 1 | P.N. | R0 | Absent | 15% + | 30 | Desert | 2 NED |
| 46 | M | 50 | 2 cm/R | pT1aNxMx | 1 | P.N. | R0 | Absent | Neg. | 0 | Desert | 2 NED |
| 47 | M | 50 | 1,5 cm/R | pT1aNxMx | 2 | P.N. | R0 | Absent | Neg. | 0 | Desert | 2 NED |
| 48 | M | 58 | 3 cm/R | pT1aNxMx | 1 | P.N. | R0 | Absent | Neg. | 0 | Desert | 2 NED |
| 49 | F | 71 | 4 cm/L | pT1aNxMx | 2 | P.N. | R0 | Absent | Neg. | 0 | Desert | 2 NED |
| 50 | F | 41 | 1,5 cm/R | pT1aNxMx | 1 | P.N. | R0 | Absent | Neg. | 0 | Inflamed | 3 NED |
| 51 | M | 53 | 4,5 cm/L | pT1bNxMx | 2 | P.N. | R0 | Absent | Neg. | 0 | Desert | 3 NED |
| 52 | M | 67 | 4,6 cm/R | pT1bNxMx | 2 | R.N. | R0 | Absent | Neg. | 0 | Desert | 3 NED |
| 53 | F | 55 | 3 cm/L | pT1aNxMx | 2 | P.N. | R0 | Absent | Neg. | 0 | Desert | 3 NED |
| 54 | M | 77 | 5,5 cm/L | pT1bNxMx | 2 | R.N. | R0 | Absent | Neg. | 0 | Desert | 3 NED |
| 55 | M | 67 | 2,6 cm/R | pT1aNxMx | 2 | P.N. | R0 | Absent | Neg. | 0 | Desert | 3 NED |
| 56 | M | 39 | 1,2 cm/L | pT1aNxMx | 2 | P.N. | R0 | Absent | Neg. | 0 | Desert | 4 NED |
| 57 | M | 39 | 3,5 cm/L | pT1aNxMx | 1 | P.N. | R0 | Absent | Neg. | 0 | Desert | 4 NED |
| 58 | F | 47 | 4,5 cm/R | pT1bNxMx | 2 | P.N. | R0 | Absent | Neg. | 0 | Desert | 4 NED |
| 59 | F | 91 | 5 cm/L | pT3aNxMx | 2 | R.N. | R0 | Absent | Neg. | 0 | Desert | 4 NED |
| 60 | M | 73 | 2 cm/L | pT1aNxMx | 2 | P.N. | R0 | Absent | 30% + | 90 | Desert | 5 NED |
| 61 | M | 62 | 3,5 cm/R | pT1aNxMx | 2 | P.N. | R0 | Absent | 50% + | 80 | Desert | 5 NED |
| 62 | M | 46 | 2,2 cm/L | pT1aNxMx | 2 | P.N. | R0 | Absent | Neg. | 0 | Desert | 5 NED |
| 63 | M | 64 | 3 cm/R | pT1aNxMx | 2 | P.N. | R0 | Absent | Neg. | 0 | Desert | 5 NED |
| 64 | F | 69 | 2 cm/L | pT1aNxMx | 2 | P.N. | R0 | Absent | Neg. | 0 | Desert | 5 NED |
| 65 | M | 58 | 3,5 cm/L | pT1aNxMx | 2 | P.N. | R0 | Absent | 20% + | 25 | Excluded | 6 NED |
| 66 | M | 58 | 2 cm/L | pT1aNxMx | 1 | P.N. | R0 | Absent | Neg. | 0 | Desert | 6 NED |
| 67 | M | 58 | 3 cm/R | pT1aNxMx | 1 | P.N. | R0 | Absent | Neg. | 0 | Desert | 6 NED |
| 68 | F | 85 | 3,5 cm/R | pT1aNxMx | 2 | R.N. | R0 | Absent | Neg. | 0 | Desert | 6 NED |
| 69 | F | 47 | 9 cm/R | pT2aNxMx | 4* | P.N. | R0 | 10% | 20% + | 35 | Desert | N.A. |
| 70 | M | 57 | 4,2 cm/L | pT1bNxMx | 4 | P.N. | R0 | Absent | 80% + | 220 | Desert | N.A. |
| 71 | M | 59 | 10,5 cm/L | pT3aN1Mx | 4* | R.N. | R0 | 30% | Neg. | 0 | Desert | 3 AWD (pancreas and lymph nodes) |
| 72 | M | 57 | 10,5 cm/L | pT3aNxM1 | 4 | R.N. | R0 | 30% | 30% + | 60 | Desert | 4 AWD (adrenal gland and urinary bladder) |
| 73 | M | 82 | 4,8 cm/L | pT3aNxMx | 3 | R.N. | R0 | Absent | 30% + | 55 | Inflamed | N.A. |
| 74 | F | 41 | 6 cm/L | pT3aNxMx | 3 | R.N. | R0 | 1% | 40% + | 45 | Desert | 4 NED |
| 75 | M | 77 | 13 cm/L | pT3aNxMx | 4* | R.N. | R0 | 1% | 90% + | 270 | Desert | 5 NED |
| 76 | F | 65 | 3,5 cm/L | pT1aNxMx | 3 | P.N. | R1 | 1% | Neg. | 0 | Excluded | 5 NED |
| 77 | M | 70 | 3,5 cm/L | pT1aNxMx | 3 | P.N. | R0 | Absent | Neg. | 0 | Desert | 6 NED |
| 78 | M | 78 | 2,5 cm/L | pT1aNxMx | 4 | P.N. | R1 | 5% | 15% + | 40 | Desert | 6 NED |
| 79 | M | 62 | 4,2 cm/R | pT1bNxMx | 3 | P.N. | R0 | Absent | Neg. | 0 | Desert | 6 NED |
| 80 | M | 59 | 7,5 cm/L | pT3aNxMx | 3 | R.N. | R0 | Absent | Neg. | 0 | Desert | 6 NED |
| 81 | M | 71 | 5 cm/R | pT1bNxM1 | 3 | P.N. | R0 | Absent | 40% + | 110 | Inflamed | 5 AWD (lung) |
| 82 | M | 63 | 7,5 cm/L | pT2aNxMx | 3 | P.N. | N.A. | Absent | Neg. | 0 | Inflamed | N.A. |
| 83 | M | 62 | 10,5 cm/R | pT3aNxMx | 3 | R.N. | R0 | Absent | Neg. | 0 | Desert | 7 NED |
| 84 | F | 73 | 5 cm/L | pT1bNxMx | 3 | P.N. | R1 | Absent | Neg. | 0 | Desert | 8 NED |
| 85 | M | 76 | 7,5 cm/L | pT2aNxMx | 3 | R.N. | R0 | Absent | Neg. | 0 | Inflamed | 8 NED |
| 86 | F | 49 | 8,5 cm/L | pT3aNxMx | 3 | R.N. | R0 | Absent | Neg. | 0 | Desert | 124 NED |
| 87 | M | 66 | 6 cm/L | pT1bNxMx | 3 | R.N. | R0 | 5% | Neg. | 0 | Inflamed | 92 NED |
| 88 | F | 32 | 5,1 cm/R | pT3aNxMx | 2 | R.N. | R0 | Absent | Neg. | 0 | Desert | 1 NED |
| 89 | M | 65 | 4 cm/R | pT3aNxMx | 4 | R.N. | R0 | Absent | Neg. | 0 | Desert | 83 Urothelial cancer on F.U. |
| 90 | M | 63 | 9,5 cm/L | pT3aNxM1 | 4 | R.N. | R0 | 1% | 80% + | 220 | Inflamed | 1 AWD (adrenal gland) |
| 91 | M | 71 | 10,5 cm/R | pT3aNxMx | 4* | R.N. | R0 | 20% | 5% + | 5 | Desert | 1 NED |
| 92 | M | 72 | 8,3 cm/R | pT3aNxMx | 4* | R.N. | R0 | 30% | Neg. | 0 | Excluded | 6 NED |
| 93 | M | 70 | 6 cm/R | pT3aNxMx | 2 | R.N. | R0 | Absent | Neg. | 0 | Desert | 1 NED |
| 94 | F | 85 | 7 cm/L | pT3aNxMx | 4 | R.N. | R0 | 1% | Neg. | 0 | Desert | 89 NED |
| 95 | M | 79 | 6,5 cm/R | pT1bNxMx | 3 | R.N. | R0 | Absent | 30% + | 50 | Desert | 1 NED |
| 96 | F | 74 | 9 cm/L | pT3aNxMx | 3 | R.N. | R0 | Absent | 30% + | 70 | Inflamed | 30 AWD (local recurrence, lung) |
| 97 | F | 63 | 6,2 cm/R | pT3aNxMx | 4 | R.N. | R0 | 10% | 40% + | 100 | Inflamed | 2 NED |
| 98 | M | 62 | 12 cm/R | pT3aNxMx | 4* | R.N. | R0 | 1% | 10% + | 30 | Desert | 97 NED |
| 99 | F | 66 | 5 cm/R | pT3aNxMx | 4* | R.N. | R0 | 40% | 5% + | 10 | Desert | 26 DOD (local recurrence) |
| 100 | F | 70 | 5,3 cm/L | pT3aNxMx | 2 | R.N. | R0 | Absent | Neg. | 0 | Inflamed | 41 NED |
| 101 | M | 56 | 6,5 cm/L | pT1bNxMx | 2 | R.N. | R0 | Absent | Neg. | 0 | Desert | 1 NED |
| 102 | F | 80 | 4,2 cm/L | pT3aNxM1 | 4 | R.N. | R0 | 25% | 10% + | 30 | Desert | 2 AWD (vagina) |
| 103 | M | 74 | 10,5 cm/L | pT3aNxMx | 3 | R.N. | R0 | 20% | 90% + | 230 | Desert | 31 AWD (lung and brain) |
| 104 | M | 67 | 3,5 cm/R | pT3aNxMx | 3 | R.N. | R0 | Absent | Neg. | 0 | Inflamed | 1 NED |
| 105 | M | 64 | 8,5 cm/R | pT2aNxMx | 3 | R.N. | R0 | 1% | Neg. | 0 | Desert | 26 AWD (liver) |
| 106 | M | 71 | 15 cm/L | pT3aNxMx | 4 | R.N. | R0 | 40% | 5% + | 10 | Inflamed | 8 DOD (lung) |
| 107 | F | 71 | 4,2 cm/R | pT1bNxMx | 3 | R.N. | R0 | Absent | 5% + | 5 | Desert | 89 NED |
| 108 | M | 49 | 4 cm/R | pT1aNxMx | 2 | R.N. | R0 | Absent | Neg. | 0 | Desert | 2 NED |
| 109 | M | 38 | 7,5 cm/L | pT3aNxMx | 2 | R.N. | R0 | Absent | Neg. | 0 | Desert | 84 NED |
| 110 | M | 76 | 10,5 cm/R | pT3aNxM1 | 3 | R.N. | R0 | 1% | Neg. | 0 | Desert | 3 DOF (adrenal gland and lung) |
| 111 | M | 66 | 6,5 cm/R | pT3aNxMx | 4 | R.N. | R0 | 10% | Neg. | 0 | Desert | 14 NED |
| 112a^§^ | M | 76 | 5,5 cm/L | pT3aNxMx | 4* | R.N. | R0 | 10% | 40% + | 100 | Desert | 10 NED |
| 112b^§^ | M | 76 | 3,5 cm/L | pT3aNxMx | 2 | R.N. | R0 | Absent | 30% + | 70 | Inflamed | 10 NED |
| 113 | F | 46 | 5 cm/L | pT3aNxMx | 3 | R.N. | R0 | Absent | Neg. | 0 | Desert | 85 NED |
| 114 | M | 77 | 5,8 cm/R | pT1bNxMx | 3 | R.N. | R0 | 1% | 20% + | 50 | Desert | 3 NED |
| 115 | F | 80 | 6 cm/L | pT3aNxMx | 3 | R.N. | R0 | Absent | Neg. | 0 | Desert | 7 NED |
| 116 | F | 45 | 9,5 cm/R | pT3aNxMx | 3 | R.N. | R0 | 20% | Neg. | 0 | Excluded | 1 NED |
| 117 | F | 80 | 4,5 cm/R | pT3aNxMx | 3 | R.N. | R0 | Absent | Neg. | 0 | Desert | 13 NED |
| 118 | M | 79 | 5,5 cm/R | pT3aNxMx | 4 | R.N. | R0 | 30% | 30% + | 50 | Desert | 61 AWD (adrenal gland and lung) |
| 119 | M | 64 | 5 cm/R | pT1bNxMx | 3 | R.N. | R0 | Absent | Neg. | 0 | Desert | 87 Prostate cancer and NHL on F.U. |
| 120 | F | 70 | 9,5 cm/L | pT3aNxMx | 4 | R.N. | R0 | 1% | Neg. | 0 | Desert | 1 NED |
| 121 | M | 56 | 12 cm/L | pT3aNxMx | 3 | R.N. | R0 | Absent | 10% + | 15 | Desert | 83 NED |
| 122 | M | 76 | 5 cm/L | pT3aNxMx | 3 | R.N. | R0 | 50% | 5% + | 10 | Desert | 10 AWD (lung and brain) |
| 123 | M | 78 | 7 cm/L | pT3aNxMx | 4 | R.N. | R0 | 40% | 90% + | 260 | Desert | 56 NED |
| 124 | F | 74 | 4,5 cm/R | pT3aNxMx | 3 | R.N. | R0 | 2% | 40% + | 105 | Desert | 1 NED |
| 125 | M | 46 | 8 cm/L | pT3aNxMx | 3 | R.N. | R0 | Absent | Neg. | 0 | Desert | 64 AWD (lung) |
| 126 | F | 71 | 4,5 cm/L | pT3aNxMx | 3 | R.N. | R0 | Absent | Neg. | 0 | Inflamed | 22 NED |
| 127 | M | 63 | 9 cm/L | pT3aNxMx | 3 | R.N. | R0 | 35% | 90% + | 250 | Desert | 6 AWD (peritoneum) |
| 128 | M | 71 | 8,5 cm/L | pT3aNxMx | 3 | R.N. | R0 | Absent | Neg. | 0 | Inflamed | 6 NED |
| 129 | F | 54 | 7,5 cm/L | pT3aNxMx | 2 | R.N. | R0 | Absent | Neg. | 0 | Desert | 1 NED |
| 130 | F | 65 | 5 cm/L | pT3aNxMx | 3 | R.N. | R0 | 10% | Neg. | 0 | Excluded | 2 NED |
| 131 | M | 52 | 7 cm/L | pT3aNxMx | 4 | R.N. | R0 | Absent | 5% + | 10 | Inflamed | 1 NED |
| 132 | M | 65 | 4,5 cm/R | pT3aNxMx | 3 | R.N. | R0 | Absent | 5% + | 10 | Inflamed | 16 NED |
| 133 | F | 60 | 11,5 cm/R | pT3aNxMx | 4 | R.N. | R0 | 50% | 10% + | 20 | Inflamed | 1 NED |
| 134 | M | 67 | 17 cm/L | pT3aNxM1 | 3 | R.N. | R0 | 1% | 60% + | 125 | Desert | 1 AWD (adrenal gland and lung) |
| 135 | M | 67 | 8,5 cm/L | pT3aNxMx | 2 | R.N. | R0 | Absent | Neg. | 0 | Desert | 51 NED |
| 136 | M | 82 | 4,5 cm/L | pT3aNxMx | 3 | R.N. | R0 | Absent | Neg. | 0 | Inflamed | 1 NED |
| 137a^§^ | M | 53 | 5 cm/R | pT3aNxMx | 3 | R.N. | R0 | Absent | 5% + | 5 | Inflamed | 2 NED |
| 137b^§^ | M | 53 | 6 cm/R | pT3aNxMx | 2 | R.N. | R0 | Absent | Neg. | 0 | Desert | 2 NED |
| 138 | M | 75 | 1,8 cm/L | pT3aNxMx | 3 | R.N. | R0 | Absent | Neg. | 0 | Desert | 65 NED |
| 139 | M | 65 | 2,5 cm/L | pT3aN1Mx | 3 | R.N. | R0 | Absent | 10% + | 10 | Desert | 12 AWD (lymph nodes) |
| 140 | M | 42 | 5 cm/R | pT3aNxMx | 3 | R.N. | R0 | Absent | Neg. | 0 | Excluded | 9 NED |
| 141 | M | 85 | 15 cm/R | pT3aNxMx | 4 | R.N. | R0 | 20% | Neg. | 0 | Excluded | 1 NED |
| 142 | M | 49 | 9 cm/R | pT3aNxMx | 2 | R.N. | R0 | Absent | Neg. | 0 | Desert | 74 NED |
| 143 | M | 60 | 5,5 cm/R | pT3aNxMx | 3 | R.N. | R0 | 1% | 20% + | 40 | Desert | 39 AWD (lung) |
| 144 | F | 69 | 5,7 cm/L | pT1bNxMx | 2 | R.N. | R0 | Absent | Neg. | 0 | Desert | 50 AWD (local recurrence) |

*presence of sarcomatoid component.

#same patient (35a renal tumor, 35b lung metastasis).

§same patient (“a” and “b” different primary tumors within the same kidney).

Abbreviations: TILs: tumor-infiltrating lymphocytes, R.N.: radical nephrectomy. P.N.: partial nephrectomy, N.A.: no data available, NED: no evidence of disease, F.U.: follow up, AWD: alive with disease, DOD: dead of disease.
